# Simulation of an Austenite-Twinned-Martensite Interface

**DOI:** 10.6028/jres.108.036

**Published:** 2003-12-01

**Authors:** A.J. Kearsley, L. A. Melara

**Affiliations:** National Institute of Standards and Technology, Gaithersburg, MD 20899-0001

**Keywords:** Austenite-Martensite interface, finite element method, quasi-Newton method

## Abstract

Developing numerical methods for predicting microstructure in materials is a large and important research area. Two examples of material microstructures are Austenite and Martensite. Austenite is a microscopic phase with simple crystallographic structure while Martensite is one with a more complex structure. One important task in materials science is the development of numerical procedures which accurately predict microstructures in Martensite. In this paper we present a method for simulating material microstructure close to an Austenite-Martensite interface. The method combines a quasi-Newton optimization algorithm and a nonconforming finite element scheme that successfully minimizes an approximation to the total stored energy near the interface of interest. Preliminary results suggest that the minimizers of this energy functional located by the developed numerical algorithm appear to display the desired characteristics.

## 1. Introduction

Simulation of microstructures is important for determining the behavior of complex materials [1,2,3,4,5,6]. Applying macroscopic loads to some materials can reveal their mechanical properties. These properties are associated with the microscopic structures created due to the material deformation. Microscopic structures of interest are often found near internal surfaces which, in turn are found in systems of liquid-liquid, solid-liquid and solid-solid boundaries [7].

**Fig. 1:**
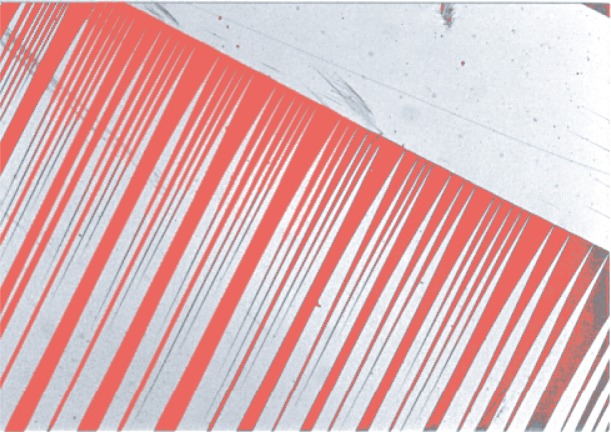
Twinned-Martensite is shown to the lower left of the diagonal line. The field of view is in the order of µm.

An *Austenite-twinned-Martensite interface* is an example of an internal surface. This interface conjoins two phases: Austenite and Martensite. Figure 1 shows an example of the twinned-Martensitic microstructures. These microscopic structures are the result of thermal or mechanical loading of the material. When a material is deformed, the structural organization of the atoms is rearranged. At the microscopic level, this rearrangement can create the crystallographic patterns shown in Figure 1, for example. These patterns represent 3-dimensional microscopic structures. There are many different ways to rearrange the atoms that make up a material and each variant corresponds to a particular arrangement. We focus on the two specific variants of Martensite distinguished by the alternating shaded areas in Figure 1.

One important mechanical property associated with an Austenite-twinned-Martensite interface is the Shape Memory Effect (SME) characteristic of Shape Memory Alloys (SMAs). SMAs are materials that, when deformed for an indefinite period of time, return to their original shape. The deformed state is a metastable state [8]. In metastability, the total stored energy is at a local minimum, thus requiring energy to induce a transformation. In SMAs, heat will trigger the SME which in turn, returns the SMA to its original shape. An Austenite-twinned-Martensite interface is observed in materials when they are in a metastable state. Therefore, to simulate Martensite, we minimize the total stored energy near an internal surface. In this paper, we present a numerical technique used to obtain a solution which represents the spatial structure of the twinned Martensite. The technique employs a $Q_{1}$ finite element discretization of a function representing total energy and a limited memory quasi-Newton method to minimize the resulting approximate total energy function. The gradient of the function is calculated by computing the partial derivatives using finite differences. The result is an apparently robust method for approximating what are usually difficult minimizers to locate.

### 1.1 Total Stored Energy

A myriad of research methods can be found which focus on the simulation of twinned Martensite, and related microstructures, e.g. Refs. [9,10,11,12,13,14]). Previous numerical work includes minimization of the total stored energy using the conjugate gradient methods [15], the method of steepest descent [15], and a descent method [16]. Further references using and studying the use of finite element methods to simulate Martensitic microstructures can be found in [17,18,19,20,21,22,23,24]. We are interested in simulating the microstructures in the metastable state, thus our work will focus on the static problem.

The functional representing the total stored energy is taken from work by Kohn-Müller in [10,11]. The Martensite region is represented by the domain *Ω* = (0, *L*) × (0, *K*). Let *x* = (*x*, *y*) ∈ *Ω* ⊂ ℝ^2^, *u*: ℝ^2^ → ℝ and *u* = *u*(*x*, *y*). The double-well energy function is given by

$$J(u)=\int_{\Omega }{\left(\partial_{x}u\right)}^{2}+{\left({\left(\partial_{y}u\right)}^{2}-1\right)}^{2}dx+\varepsilon \int_{\Omega }|\partial_{yy}u|dx$$ (1)

where *u* equals 0 at *x* = 0. The *x* = 0 boundary corresponds to the internal surface. The function *u* = 0 at *x* = 0, due to the constraint of elastic compatibility [10,11]. The first integral is the elastic energy and the last integral is the surface energy. We seek a function $u\in W^{1,4}(\Omega )$ which minimizes Eq. (1) where

$$\begin{array}{c} W^{1,4}(\Omega )=\{v|v\in L^{4}(\Omega ),\partial_{x}v\in L^{4}(\Omega ),\partial_{y}v\in L^{4}(\Omega )\\ \text{and}\;v=0\;\text{at}\;x=0.\} \end{array}$$

### 1.2 Elastic Energy

The 2-D scalar model of elastic energy, in Eq. (1) is

$$\int_{\Omega }\left[{\left(\partial_{x}u\right)}^{2}+{\left({\left(\partial_{y}u\right)}^{2}-1\right)}^{2}\right]dx.$$ (2)

This term is minimized by a function *u* such that

$$\nabla u=\left(\begin{array}{c} 0\\ \pm 1 \end{array}\right).$$ (3)

**Fig. 2:**
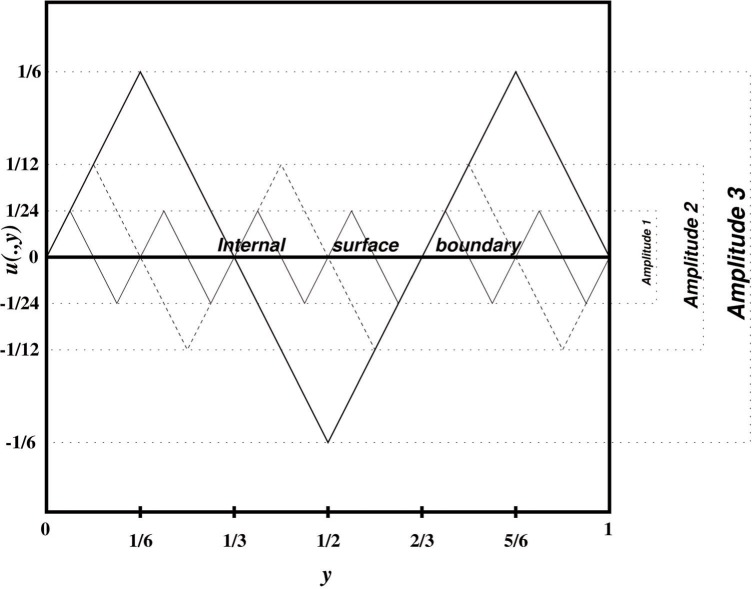
Oscillatory behavior of minimizer *u*.

Each of the gradients in Eq. (3) corresponds to a stress-free state in one of the two distinct variants of Martensite [10,11]. Examples of functions satisfying Eq. (3) are plotted in Fig. 2.

In Fig. 2 functions are piecewise linear in the *y*-direction and constant along the *x*-direction. Requiring that *u* equal zero at *x* = 0 creates more oscillations. This is similar to the behavior of Martensite near an internal surface. The function with Amplitude 1 is closest to zero, on average, than those with Amplitudes 2 and 3.

The number of oscillations is directly related to the number of discontinuities in ∂*_y_u*. We note that the function with Amplitude 1 also has the largest number of discontinuities, occurring at the peaks of *u*, where ∂*_y_u* changes from +1 to −1 (or vice-versa). Therefore, a desirable minimizer *u* has the characteristics of the function with Amplitude 1. The consequence of *u* satisfying Eq. (2) and *u* = 0 at *x* = 0 is the occurrence of more discontinuities in ∂*_y_u*. At the minimizer *u*, however, the surface energy penalizes these discontinuities.

### 1.3 Surface Energy

We employ the surface energy presented by Kohn and Müller in Ref. [10,11],

$$\varepsilon \int_{\Omega }\left|\partial_{yy}u\right|dx,$$ (4)

where ε is a constant.

The definition proposed by Kohn and Müller replaces the integral of Eq. (4) in the *y*-direction with the total variation of ∂*_y_u* over (0, *K*) [10,11]. This change designates the role of the surface energy at the minimizer *u* as a “counter” because $\frac{1}{2}\int_{0}^{K}\left|\partial_{yy}u\right|dy$ counts the number of discontinuities in ∂*_y_u*, where ∂*_y_u* ∈ {±1}. Thus, at the minimizer *u* of Eq. (1), the surface energy Eq. (4) exhibits opposite behavior to Eq. (2) since we are minimizing Eq. (1). To illustrate this counting role of Eq. (4), we briefly review functions of bounded variation.

### 1.4 Functions of Bounded Variation

The definition of functions of bounded variation is taken from Refs. [25,26,27]. Let

$$P=\{y_{0},y_{1},\ldots ,y_{l}\}$$

be any partition of (0, *K*) and let

$$\left|P\right|=\underset{j}{\max}\left|y_{j+1}-y_{j}\right|.$$

The partitioning P is a collection of points *y_j_*, *j* = 0, 1, …, *l*, such that *y*_0_ = 0 and *y_l_* = *K*. For each partitioning P, let

$$S_{P}(\bar{x})=\sum_{j=0}^{l=1}\left|\partial_{y}u\left(\bar{x},y_{j+1}\right)-\partial_{y}u\left(\bar{x},y_{j}\right)\right|,$$ (5)

for a fixed $\bar{x}\in (0,L)$. The *total variation* of ∂*_y_u* over (0, *K*) is

$$\underset{P}{\sup}S_{P}(\bar{x}),$$ (6)

where the supremum is taken over all countable partitions *P* of (0, *K*). If $0\leq S_{P}(\bar{x})\leq +\infty$, then 0 ≤ *ε* ∫*_Ω_* | ∂*_yy_u* | d*x* ≤ +∞. If *ε* ∫*_Ω_* | ∂*_yy_u* | d*x* < +∞, then ∂*_y_u* is a function of *bounded variation*.

Next, we describe the substitution of $\int_{0}^{K}\left|\partial_{yy}u\right|dy$ by Eq. (6). We assume that ∂*_y_u* ∈ *C*^1^(Ω). Using Eq. (5) and the Mean Value Theorem, we have for some *ζ_j_* ∈ (*y_j_*, *y_j_*_+1_), *j* = 1, 2, …, *l* that

$$S_{P}(\bar{x})=\sum_{j=0}^{l-1}\left|\partial_{y}u\left(\bar{x},y_{j+1}\right)-\partial_{y}u\left(\bar{x},y_{j}\right)\right|$$ (7)

$$=\sum_{j=0}^{l-1}\left|\partial_{yy}u\left(\bar{x},\varsigma_{j}\right)\right|\left(y_{j+1}-y_{j}\right),$$ (8)

and we obtain by Theorem 2.9 in Ref. [25]:

$$\begin{array}{c} \underset{P}{\sup}S_{P}(\bar{x})=\underset{|P|\to 0}{\lim}S_{P}(\bar{x})=\underset{|P|\to 0}{\lim}\sum_{j=0}^{l-1}\left|\partial_{yy}u\left(\bar{x},\varsigma_{j}\right)\right|\left(y_{j+1}-y_{j}\right)\\ =\int_{0}^{K}\left|\partial_{yy}u\right|dy \end{array}$$ (9)

**Fig. 3:**
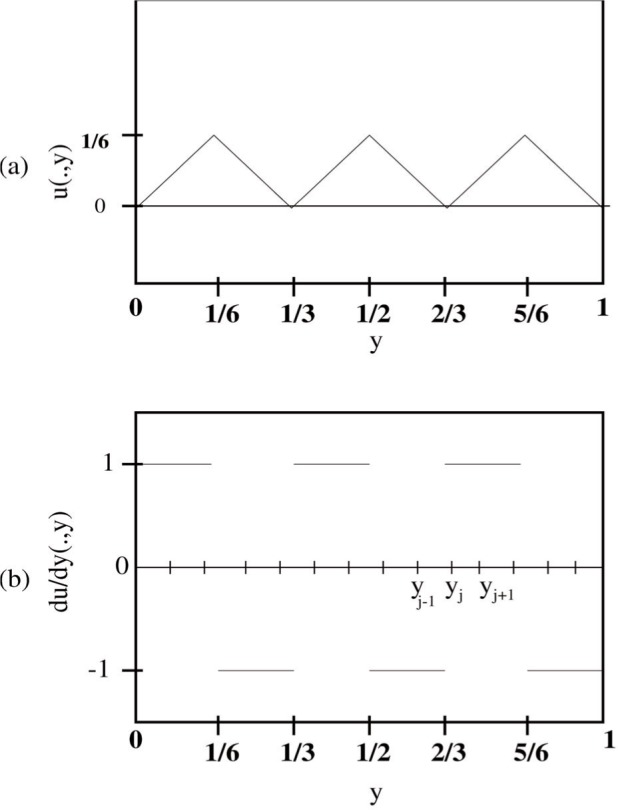
Surface energy term as a counter. Pictorial example illustrating the role of surface energy $\int_{0}^{1}\left|\partial_{yy}u\right|dy$ as a counter term. The notation *u*(·, *y*) means that the graph shows the *y*-dependence only.

The assumption that *u* ∈ *C*^1^(Ω) is stronger than the assumtion that $u\in W^{1,4}(\Omega )$; however, the equivalence in Eq. (9) is possible since the changes in ∂*_y_u* do not occur in subintervals of measure zero, [9,10,11,28]. Figure 3 shows that $\frac{1}{2}\int_{0}^{K}\left|\partial_{yy}u\right|dy$ counts the number of sign changes in ∂*_y_u*. Let (0, *K*) = (0, 1).

In Fig. 3a, the piecewise linear function $u(\bar{x},y)$ is plotted over (0, 1). In Fig. 3b, the function $\partial_{y}u(\bar{x},y)$ is plotted over (0, 1). Clearly, the number of discontinuities in ∂*_y_u* is 5 from Fig. 3b. For a discontinuity at the partition point *y_j_*, we adopt the following convention:

$$\partial_{y}u(\bar{x},y_{j})\equiv \underset{y\to y_{j},y>y_{j}}{\lim}\frac{u(\bar{x},y)-u(\bar{x},y_{j})}{y-y_{j}}.$$ (10)

Now consider (*y_j_*_−1_, *y_j_*_+1_) = (*y_j_*_−1_, *y_j_*) ∪ (*y_j_*, *y_j_*_+1_) in Fig. 3b. Evaluating at the endpoint of the two subintervals gives

$$\left|\partial_{y}u(\bar{x},y_{j})-\partial_{y}u(\bar{x},y_{j-1})\right|=\left|1-(-1)\right|=2\;\text{and}$$ (11)

$$\left|\partial_{y}u(\bar{x},y_{j+1})-\partial_{y}u(\bar{x},y_{j})\right|=\;\left|1-1\right|\;=0.$$ (12)

A jump occurred in (*y_j_*_−1_, *y_j_*) because ∂*_y_u* is discontinuous at a point in this subinterval. Based on our conventional definition of $\partial_{y}u(\bar{x},y_{j})$ at a point *y_j_* in Eq. (10), we say a “jump” occurs at *y_j_* if ∂*_y_u* is discontinuous at this point. This “jump” refers to the change in values of ∂*_y_u* at *y_j_* from +1 to −1 or vice-versa.

This jump gives the nonzero value in Eq. (11). In the subinterval (*y_j_*, *y_j_*_+1_), no jump occurs in ∂*_y_u*, therefore the difference in Eq. (12) is zero. We compute Eq. (9):

$$\int_{0}^{1}\left|\partial_{yy}u(\bar{x},y)\right|dy=2\times 5=10,$$

where the magnitude of the jump is 2 and the number of discontinuities is 5. Table 1 shows the number of discontinuities for the functions presented in Fig. 2.

Therefore, we have:

$$\int_{0}^{1}\left|\partial_{yy}u(\bar{x},y)\right|dy=2\times (\text{number of discontinuities of}\;\partial_{y}u),$$

and the surface energy succeeds in serving as a “counter.” The next section describes the numerical approximation to Eq. (1).

## 2. Description of Method

**Fig. 4:**
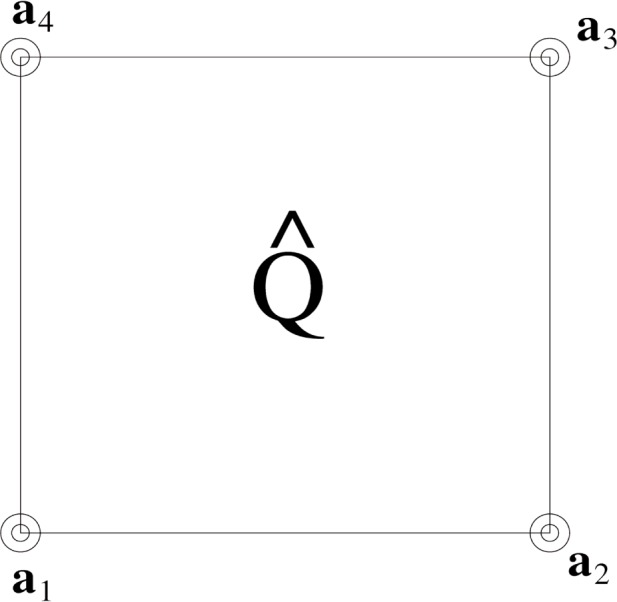
Parent Element $\hat{Q}$ with four degrees of freedom labeled *u*(**a**_1_), *u*(**a**_2_), *u*(**a**_3_), and *u*(**a**_4_).

The total stored energy Eq. (1) consists of elastic plus surface energy. First, we describe the implementation of the elastic energy followed by a discussion of the surface energy implementation. We computed Eq. (2) using affine finite elements, [29]. We chose the finite element space:

$$Q_{1}=\text{span}\{1,x,y,xy\},$$ (13)

because functions in this polynomial space lie in $W^{1,4}(\Omega )$, which satisfy the criteria in Eq. (3) for a minimizer *u* of Eq. (1). The elements are rectangles with the function values at the vertices as degrees of freedom. The parent element is $\hat{Q}=(0,1)\times (0,1)$ and for some *k* ∈ ℕ, the reference element is

$$Q_{k}=\left(ih_{1},(i+1)h_{1}\right)\times \left(jh_{2},(j+1)h_{2}\right)\subset \Omega =\underset{P}{\cup }Q_{P},$$

where *h*_1_ is the mesh size along the *x*-axis and *h*_2_ is the mesh size along the *y*-axis. The degrees of freedom of the elements are the function values at the vertices, and for $\hat{Q}$ we label them *u*(**a**_1_), *u*(**a**_2_), *u*(**a**_3_), and *u*(**a**_4_). Figure 4 shows the parent element with the vertices also denoted by concentric circles.

### 2.1 Affine Finite Elements

Let *F*: $\hat{Q}\to Q_{k}$ be given by

$$F\left(\begin{array}{c} \hat{x}\\ \hat{y} \end{array}\right)=\left(\begin{array}{cc} h_{1} & 0\\ 0 & h_{2} \end{array}\right)\left(\begin{array}{c} \hat{x}\\ \hat{y} \end{array}\right)+\left(\begin{array}{c} ih_{1}\\ jh_{2} \end{array}\right).$$ (14)

We simplify, by letting $F=F(\hat{x},\hat{y})$. We use the affine map Eq. (14) to evaluate the function *u*, to compute ∇*u* and to integrate. The function *u* is spanned by the four basis functions defined in each element *Q_k_*. Using Eq. (14), we can determine all basis functions by the four basis functions in $\hat{Q}$. Thus, the approximation to *u* is:

$$u(x,y)=u_{1}\phi_{1}(\hat{x},\hat{y})+u_{2}\phi_{2}(\hat{x},\hat{y})+u_{3}\phi_{3}(\hat{x},\hat{y})+u_{4}\phi_{4}(\hat{x},\hat{y}),$$ (15)

where $x=\hat{x}(x,y)$, $y=\hat{y}(x,y)$. The *n*th basis function in *Q* is

$$\phi_{n}(\hat{x},\hat{y})=\alpha_{0}^{\left(n\right)}+\alpha_{1}^{\left(n\right)}\hat{x}+\alpha_{2}^{\left(n\right)}\hat{y}+\alpha_{3}^{\left(n\right)}\hat{x}\hat{y},$$

and it satisfies the property that *ϕ_n_*(**a***_m_*) = *δ_mn_*, for *n*, *m* = 1, 2, 3, 4, where *δ_mn_* is the Kronecker delta function.

To compute the gradient of *u* and to integrate *u*, we need: ∇*F*, det∇*F* and ∇*F*^−1^. The gradient of *F* is

$$\nabla F=\left(\begin{array}{cc} h_{1} & 0\\ 0 & h_{2} \end{array}\right)$$ (16)

and, we have

$$\nabla \phi_{n}(x,y)=\nabla F^{-1}\nabla \phi_{n}(\hat{x},\hat{y}).$$ (17)

Using Eqs. (15), (16), and (17) we have

$$\nabla u(x,y)=\nabla F^{-1}\sum_{n=1}^{4}u_{n}\nabla \phi_{n}(\hat{x},\hat{y}).$$

To integrate the elastic energy over *Q_k_* we use Eqs. (15)–(17) and obtain

$$\begin{array}{c} \int_{Q_{k}}{\left(\partial_{x}u\right)}^{2}dxdy=\int_{\hat{Q}}{\left(\frac{1}{h_{1}}\sum_{n=1}^{4}u_{n}\partial_{\hat{x}}\phi_{n}(\hat{x},\hat{y})\right)}^{2}\;\det\;\nabla Fd\hat{x}d\hat{y},\\ =\int_{\hat{Q}}{\left(\frac{1}{h_{1}}\sum_{n=1}^{4}u_{n}\partial_{\hat{x}}\phi_{n}(\hat{x},\hat{y})\right)}^{2}\;h_{1}h_{2}d\hat{x}d\hat{y}. \end{array}$$ (18)

The procedure is the same for

$$\int_{Q_{k}}{\left({\left(\partial_{y}u\right)}^{2}-1\right)}^{2}dxdy.$$

### 2.2 Computation of Surface Energy

We evaluate the surface energy term in Eq. (4) using Eq. (6), approximating *∂_y_u* using backward finite differences,

$$\partial_{y}u(\bar{x},y_{j})\approx \frac{u(\bar{x},y_{j})-u(\bar{x},y_{j-1})}{h_{2}}$$

and

$$\partial_{y}u(\bar{x},y_{j+1})\approx \frac{u(\bar{x},y_{j+1})-u(\bar{x},y_{j})}{h_{2}}.$$

Here,

$$\int_{0}^{K}\left|\partial_{yy}u\right|dy=\sum_{j=0}^{N(h_{2})-1}\left|\partial_{y}u(\bar{x},y_{j+1})-\partial_{y}u(\bar{x},y_{j})\right|$$ (19)

where *N*(*h*_2_) is the number of nodes along the *y*-direction. We can integrate along the *x*-direction to complete the approximation of Eq. (4).

## 3. Numerical Implementation

The computation of the integral in Eq. (18) is done exactly in the parent element $\hat{Q}$. The symbolic package Maple^1^ [30] was employed to integrate this term exactly. The algebraic expression consisting of nodes *u_j_* and mesh sizes *h*_1_ and *h*_2_, is evaluated over each element.

The gradient of Eq. (1) is approximated using cell-centered finite differences. Let $u\in W^{1,4}(\Omega )$. The Gateaux derivative is:

$$G(u)=\underset{t\to 0}{\lim}\frac{1}{t}\left[J(u+t\varphi )-J(u)\right]$$ (20)

where $\varphi \in W^{1,4}(\Omega )$ [31]. The linear operation *G* is the directional derivative of *J* in the direction *φ*. From the Euler-Lagrange equations, we see that the gradient of *J* is difficult to compute. Considering the Euler-Lagrange equation we have,

$$\begin{array}{l} G(u)=\frac{d}{dt}J(u+t\varphi ){|}_{t=0}=\int_{\Omega }\frac{\partial J(u)}{\partial \nabla u}\nabla \varphi dx,\\ \;=-\int_{\Omega }\text{div}\frac{\partial J}{\partial \nabla u}\varphi dx, \end{array}$$

where the gradient is div (∂*J*/∂∇*u*). Let *J*(u) be the discretized representation of the total energy Eq. (1) for u ∈ ℝ*^N^*^(^*^h^*^)^, where *N*(*h*) is the total number of nodes in Ω.

With this numerical approximation to ∇*J*(u), we use the limited memory BFGS method [32] to numerically minimize *J*(u). This inexpensive quasi-Newton method seeks to build an approximation *H*^(^*^k^*^)^ to the inverse of the Hessian matrix of second derivatives of *J* at the point u^(^*^k^*^)^. These inexpensive approximations are constructed from a small number of vectors updated at every iteration.

The *k* + 1 step of the limited memory BFGS method is given by:

$$u^{\left(k+1\right)}=u^{\left(k\right)}-\alpha^{\left(k\right)}H^{\left(k\right)}\nabla J\left(u^{\left(k\right)}\right).$$

where the parameter *α*^(^*^k^*^)^ is a line search parameter and is computed at each iteration using a line-search procedure.

The Hessian matrix *B*^(^*^k^*^)^ satisfies the secant equation:

$$B^{\left(k\right)}s^{\left(k\right)}=y^{\left(k\right)},$$

where

$$\begin{array}{l} s^{\left(k\right)}=\left(u^{\left(k\right)}-u^{\left(k-1\right)}\right),\;\text{and}\\ y^{\left(k\right)}=\nabla J\left(u^{\left(k\right)}\right)-\nabla J\left(u^{\left(k-1\right)}\right). \end{array}$$

We employ a limited memory BFGS method because the Hessian matrix *B*^(^*^k^*^)^ is expensive to compute since we are solving a large scale optimization problem [32,33]. This is done by storing a certain number of vector pairs {s^(^*^k^*^)^, *y*^(^*^k^*^)^}. At each new iterate, the oldest vector pair is deleted and replaced by the new vector pair, thus preserving curvature information only from the most recent iterations. The formula for updating the inverse of the Hessian matrix *H*^(^*^k^*^)^ = (*B*^(^*^k^*^)^)^−1^ is:

$$\begin{array}{l} H^{\left(k+1\right)}={\left(V^{\left(k-m\right)}\ldots V^{\left(k-1\right)}\right)}^{T}{\left(H^{\left(k\right)}\right)}^{0}\left(V^{\left(k-m\right)}\ldots V^{\left(k-1\right)}\right)\\ +\rho^{\left(k-m\right)}{\left(V^{\left(k-m+1\right)}\ldots V^{\left(k-1\right)}\right)}^{T}s^{\left(k-m\right)}{\left(s^{\left(k-m\right)}\right)}^{T}\left(V^{\left(k-m+1\right)}\ldots V^{\left(k-1\right)}\right)\\ +\ldots \\ +\rho^{\left(k-1\right)}s^{\left(k-1\right)}{\left(s^{\left(k-1\right)}\right)}^{T}. \end{array}$$

where ${\left(H^{\left(k\right)}\right)}^{0}$ is some initial Hessian approximation,

$$V^{\left(k\right)}=I-\rho^{\left(k\right)}y^{\left(k\right)}{\left(s^{\left(k\right)}\right)}^{T},$$

and

$$\rho =\frac{1}{{\left(y^{\left(k\right)}\right)}^{T}s^{\left(k\right)}}.$$

## 4. Results

**Fig. 5:**
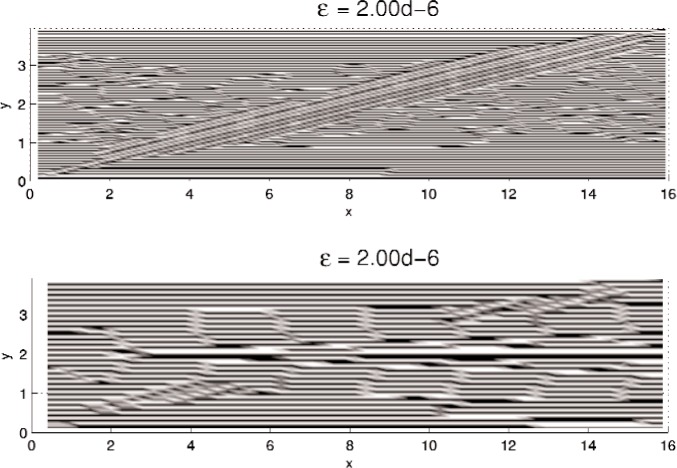
Comparison of density plots of *u* for values of *ε* = 2 × 10^−6^ on 60 × 60 grid (bottom) vs 120 × 120 grid (top).

**Fig. 6:**
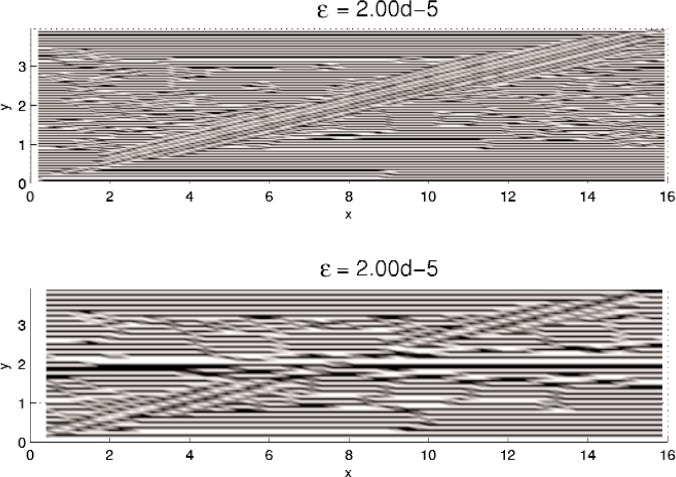
Comparison of density plots of *u* for values of *ε* = 2 × 10^−5^ on 60 × 60 grid (top) vs 120 × 120 grid (bottom).

**Fig. 7:**
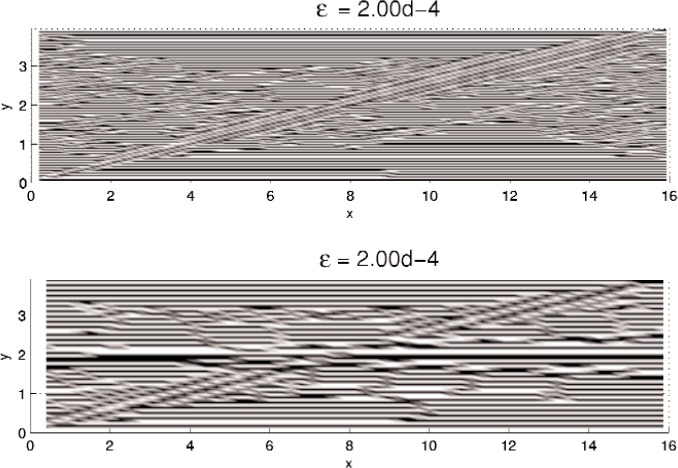
Comparison of density plots of *u* for values of *ε* = 2 × 10^−4^ on 60 × 60 grid (top) vs 120 × 120 grid (bottom).

**Fig. 8:**
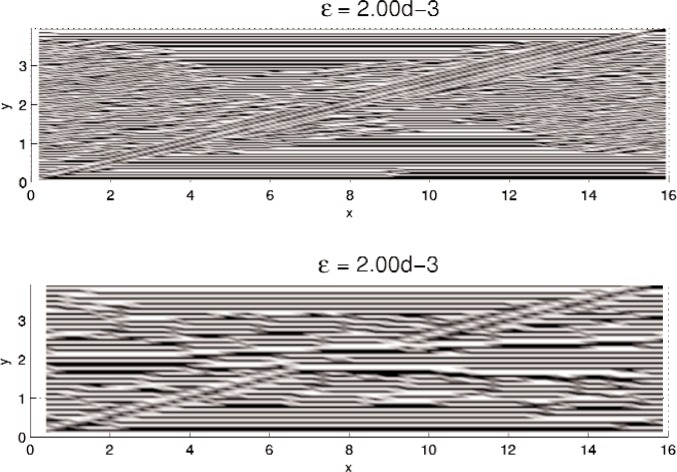
Comparison of density plots of *u* for values of *ε* = 2 × 10^−3^ on 60 × 60 grid (top) vs 120 × 120 grid (bottom).

**Fig. 9:**
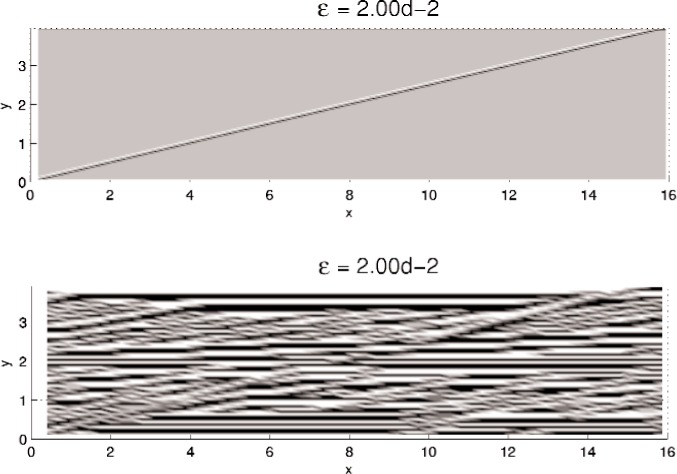
Comparison of density plots of *u* for values of *ε* = 2 × 10^−2^ on 60 × 60 grid (top) vs 120 × 120 grid (bottom).

**Fig. 10:**
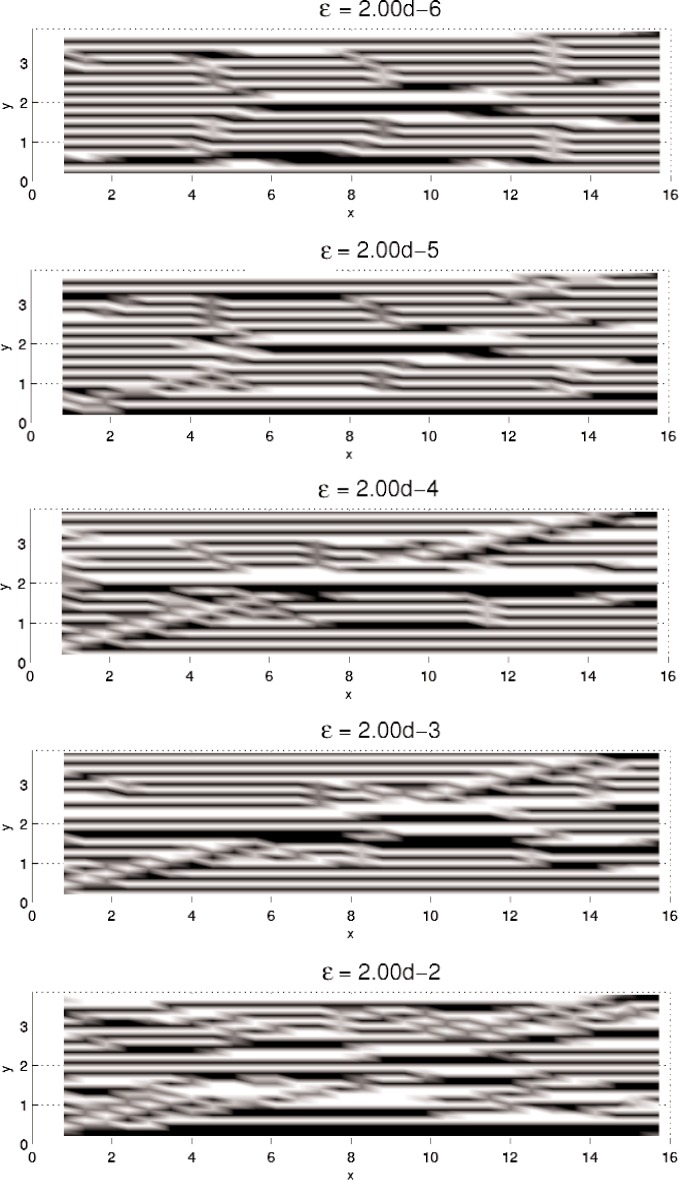
Density plot of solution *u* for *ε* = 2 × 10^−6^, …, 2 × 10^−2^ on 30 × 30 grid.

**Fig. 11:**
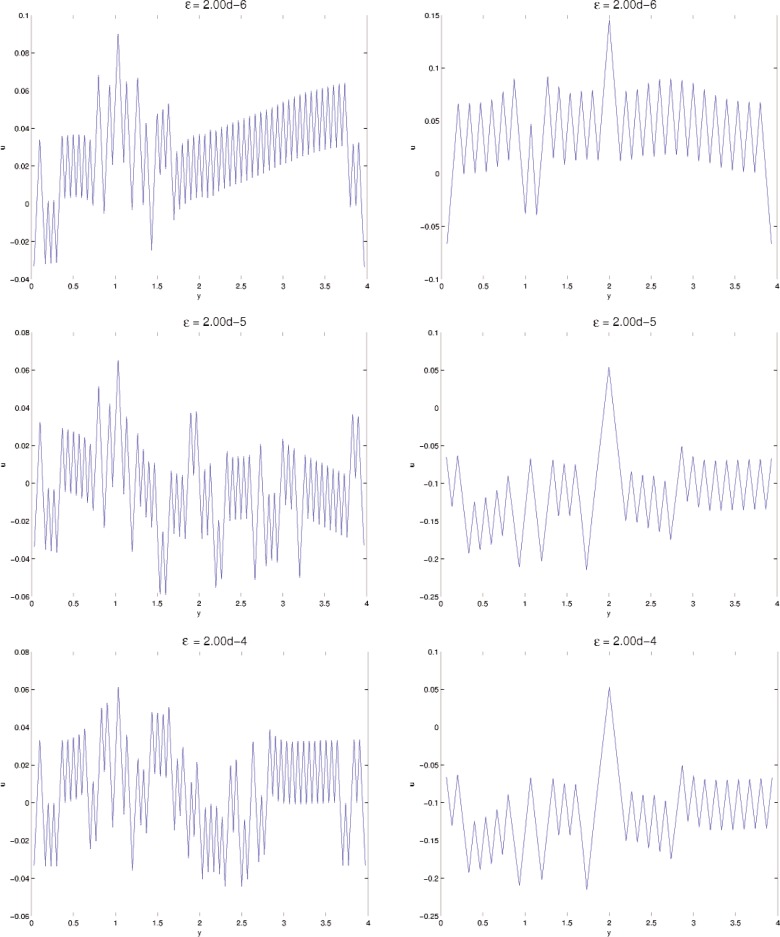
Comparison of *u*(4, *y*) for various values of *ε* = 2 × 10^−6^, …, 2 × 10^−4^ on 120 × 120 (left column) grid vs 60 × 60 grid (right column).

**Fig. 12:**
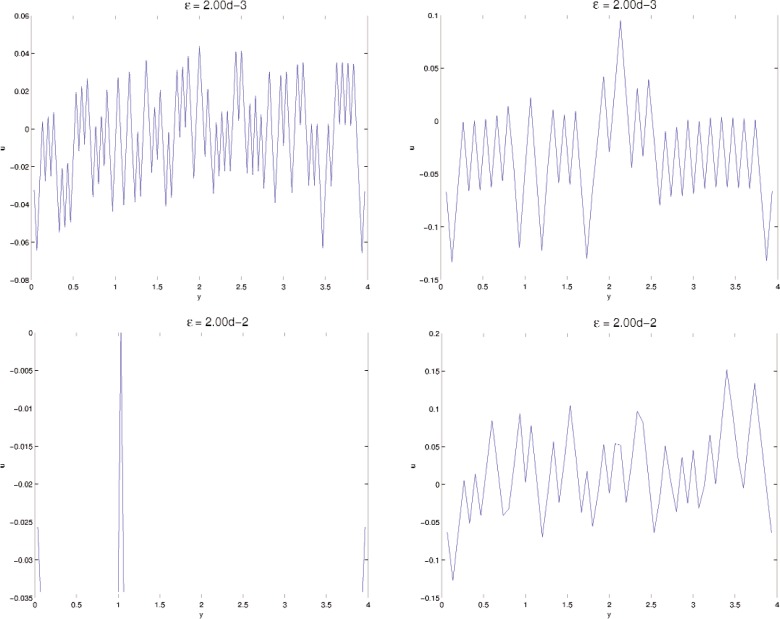
Comparison of *u*(4, *y*) for various *ε* = 2 × 10^−3^ and *ε* = 2 × 10^−2^ on 120 × 120 grid (left column) vs 60 × 60 grid (right column).

**Fig. 13:**
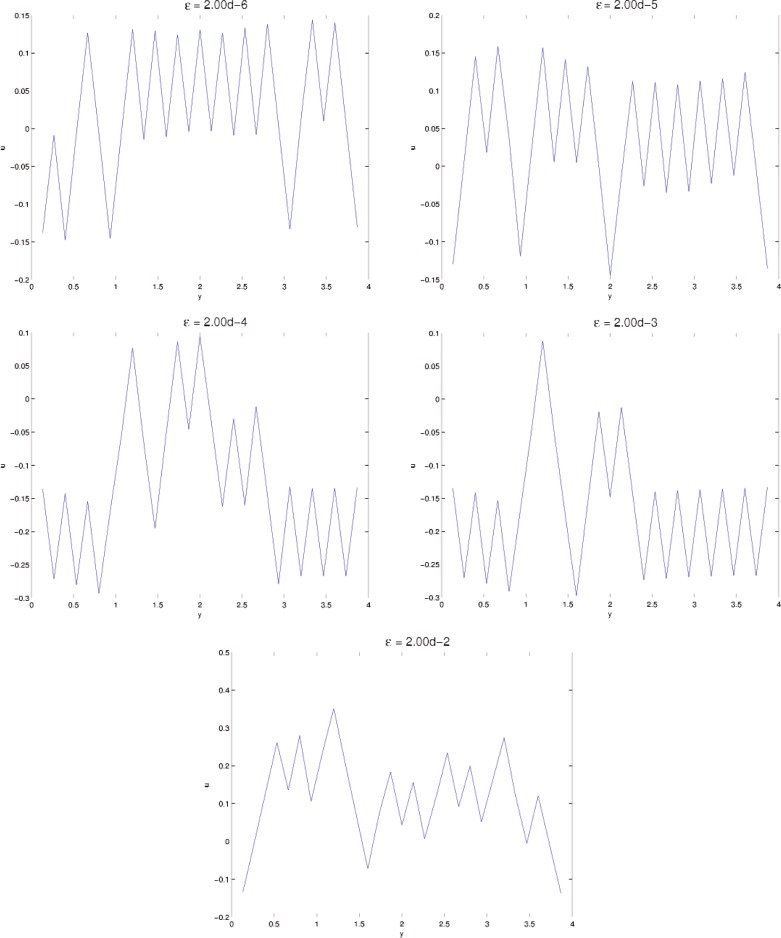
Profiles of *u*(4, *y*) for *ε* = 2 × 10^−6^, …, 2 × 10^−2^ on a 30 × 30 grid.

In this section, results computed on the various grids, 30 × 30, 60 × 60, and 120 × 120, are presented. In all minimization, the initial iterate **u**^0^ is a small (≈10^−3^) perturbation of pure Austenite, which we take to be *u* = 0. Density plots of the minimizer *u* are shown in Figs. 5–10 and demonstrate the existence of the desired microstructures in the twinned-Martensite phase. These figures also demonstrate the role of the surface energy as a penalization term of Eq. (1). We seem to be able to control the number of microstructures by varying the values of *ε*, with a larger value of *ε* resulting in a smaller number of microstructures in Martensite and vice-versa. Figures 11, 12, and 13 show plots of the profiles of minimizer *u* at *x* = 4. Figures 11, 12, and 13 exhibit a larger number of discontinuities in ∂*_y_u* for *ε* = 2 × 10^−6^ than for *ε* = 2 × 10^−2^. In Figs. 5–9 one sees the effect of grid-size comparing results on 60 × 60 grid with 120 × 120 grid for *ε* = 2 × 10^−6^ through *ε* = 2 × 10^−2^. The black and white stripes correspond directly to the saw-toothed behavior of *u* at *x* = 4. For small *ε* values, the number of discontinuities is close to the number of partitions along the *y*-direction. Tables 2–4 show the energy values for the minimizers shown in Figs. 5–10. The column with *E*(*u*) corresponds to the elastic energy values of the minimizer while the column with *S*(*u*) corresponds to the surface energy values of the minimizer. The energy values presented in Tables 3 and 4 appear consistent with these conclusions. The larger the *ε*, the larger the value of the surface energy at the minimizer. In Table 3 the value of the surface energy increases by a larger order than that of the elastic energy.

One final and curious observation is the appearance of a “diagonal band” structure in Figs. 5–10. It would seem that this band, which is apparent for solutions on various grid sizes, is related to the competing roles of the surface and elastic energies is and may be associated to the “equipartitioning of energy” principle proposed in Kohn and Müller in Ref. [10,11]. Future research plans include a deeper investigation of this diagonal-band structure.

## Figures and Tables

**Table 1:** Number of “jumps” in ∂*_y_ u*

Function	$\int_{0}^{1}\left|\partial_{yy}u\right|dy$	Discontinuities
Amplitude 1	24	12
Amplitude 2	12	6
Amplitude 3	6	3

**Table 2:** Energy values for various *ε* on 120 × 120 grid

*ε*	*E*(*u*)	*ε S*(*u*)	Total energy
2 × 10^−6^	6.3014	0.5691e-2	6.3071
2 × 10^−5^	6.1122	0.5698e-1	6.1692
2 × 10^−4^	7.0859	0.5474	7.6334
2 × 10^−3^	8.2222	4.5200	12.7423
2 × 10^−2^	23.3218	1.2559	24.5770

**Table 3:** Energy values for various *ε* on 60 × 60 grid

*ε*	*E*(*u*)	*ε S*(*u*)	Total energy
2 × 10^−6^	4.3684	0.3120e-2	4.3716
2 × 10^−5^	6.3005	0.2808e-1	6.3286
2 × 10^−4^	6.3010	0.2798	6.5809
2 × 10^−3^	5.7738	2.7356	8.5095
2 × 10^−2^	3.7945	14.6825	18.477

**Table 4:** Energy values for various *ε* on 30 × 30 grid

*ε*	*E*(*u*)	*ε S*(*u*)	Total energy
2 × 10^−6^	4.6093	0.0015	4.6108
2 × 10^−5^	4.9832	0.0145	4.9977
2 × 10^−4^	6.1073	0.1326	6.1499
2 × 10^−3^	5.5335	1.3246	6.8581
2 × 10^−2^	8.0650	9.6770	17.7420

## Footnotes

1 Certain commercial equipment, instruments, or materials are identified in this paper to foster understanding. Such identification does not imply recommendation or endorsement by the National Institute of Standards and Technology, nor does it imply that the materials or equipment identified are necessarily the best available for the purpose.
